# Arrestin Facilitates Rhodopsin Dephosphorylation *in Vivo*

**DOI:** 10.1523/JNEUROSCI.0141-22.2022

**Published:** 2022-04-27

**Authors:** Chia-Ling Hsieh, Yun Yao, Vsevolod V. Gurevich, Jeannie Chen

**Affiliations:** ^1^Ziliha Neurogenetic Institute, Department of Physiology and Neuroscience, Keck School of Medicine, University of Southern California, Los Angeles, California 90089; ^2^Department of Pharmacology, Vanderbilt University, Nashville, Tennessee 37232

**Keywords:** arrestin, dephosphorylation, GPCR, PP2A, rhodopsin

## Abstract

Deactivation of G-protein-coupled receptors (GPCRs) involves multiple phosphorylations followed by arrestin binding, which uncouples the GPCR from G-protein activation. Some GPCRs, such as rhodopsin, are reused many times. Arrestin dissociation and GPCR dephosphorylation are key steps in the recycling process. *In vitro* evidence suggests that visual arrestin (ARR1) binding to light-activated, phosphorylated rhodopsin hinders dephosphorylation. Whether ARR1 binding also affects rhodopsin dephosphorylation *in vivo* is not known. We investigated this using both male and female mice lacking ARR1. Mice were exposed to bright light and placed in darkness for different periods of time, and differently phosphorylated species of rhodopsin were assayed by isoelectric focusing. For WT mice, rhodopsin dephosphorylation was nearly complete by 1 h in darkness. Surprisingly, we observed that, in the *Arr1* KO rods, rhodopsin remained phosphorylated even after 3 h. Delayed dephosphorylation in *Arr1* KO rods cannot be explained by cell stress induced by persistent signaling, since it is not prevented by the removal of transducin, the visual G-protein, nor can it be explained by downregulation of protein phosphatase 2A, the putative rhodopsin phosphatase. We further show that cone arrestin (ARR4), which binds light-activated, phosphorylated rhodopsin poorly, had little effect in enhancing rhodopsin dephosphorylation, whereas mice expressing binding-competent mutant ARR1-3A showed a similar time course of rhodopsin dephosphorylation as WT. Together, these results reveal a novel role of ARR1 in facilitating rhodopsin dephosphorylation *in vivo*.

**SIGNIFICANCE STATEMENT** G-protein-coupled receptors (GPCRs) are transmembrane proteins used by cells to receive and respond to a broad range of extracellular signals that include neurotransmitters, hormones, odorants, and light (photons). GPCR signaling is terminated by two sequential steps: phosphorylation and arrestin binding. Both steps must be reversed when GPCRs are recycled and reused. Dephosphorylation, which is required for recycling, is an understudied process. Using rhodopsin as a prototypical GPCR, we discovered that arrestin facilitated rhodopsin dephosphorylation in living mice.

## Introduction

G-protein-coupled receptors (GPCRs) are a large family of transmembrane receptors that cells use to communicate with the environment (Weis and Kobilka, 2018; Wootten et al., 2018; Smith, 2021). Receptor phosphorylation and arrestin binding are key steps in shaping and terminating GPCR signaling (Gurevich and Gurevich, 2019; Sun and Kim, 2021). Rhodopsin is a prototypical GPCR expressed by rod photoreceptors. It is rendered light-sensitive by 11-*cis* retinal that is covalently linked to the protein moiety and held within the core of the transmembrane helical bundle. Photon absorption by 11-*cis* retinal converts it to all-trans retinal, inducing a conformational change within the protein moiety (R*) (Hofmann and Palczewski, 2015). R* then initiates a phototransduction cascade that involves activation of the visual G-protein, transducin, which then activates phosphodiesterase (PDE6) to degrade cGMP, leading to closure of the cGMP-gated channels at the plasma membrane (Pugh and Lamb, 1993). Rhodopsin deactivation is initiated by the addition of phosphates at its carboxyl-terminal cluster of serine and threonine residues by G-protein-coupled receptor kinase 1 (GRK1) (Wilden et al., 1986; Chen et al., 1995; Arshavsky, 2002). Phosphorylated, light-activated rhodopsin (R*-P) exhibits lower catalytic activity in transducin activation, and deactivation is completed on arrestin-1 (ARR1) binding (Xu et al., 1997). The multiple phosphorylations and ARR1 binding contribute to the reproducibility of the single-photon response (Mendez et al., 2000; Doan et al., 2006). This is an important attribute for rods since single-photon responses constitute a large proportion of the rod's operational range. Although rhodopsin activation and deactivation steps are well defined, how R*-P returns to its basal state (R) is less understood.

Unlike many GPCRs, rhodopsin is not recycled by endocytosis. Instead, new rhodopsin is synthesized in the inner segment and incorporated into nascent disks at the base of the outer segment while older discs are displaced apically until the oldest discs at the apical tip are phagocytosed by the retinal pigment epithelium (RPE) (Young, 1967). Rhodopsin lifetime is ∼10 d in mice (Nguyen-Legros and Hicks, 2000). Thus, R*-P needs to be recycled *in situ* to participate in phototransduction again. This involves dissociation of ARR1 from R*-P, rhodopsin dephosphorylation and incorporation of 11-*cis* retinal supplied through the visual cycle from the RPE (Kiser et al., 2014). As for the first two steps, it was observed that ARR1 binding to its high-affinity target, R*-P, inhibited dephosphorylation *in vitro* (Palczewski et al., 1989b; Azarian et al., 1995). This result is likely because of a steric interference of ARR1's binding to R*-P, thereby hindering the phosphatase's access to its substrate. Direct binding of ARR1 to rhodopsin-attached phosphates in the structure of the complex (Zhou et al., 2017) supports this notion. To see whether the presence of ARR1 delays rhodopsin dephosphorylation *in vivo*, we compared the rate of rhodopsin dephosphorylation in retinae of WT C57 mice and mice lacking ARR1 (*Arr1*^−/−^) (Xu et al., 1997). We used a light exposure protocol that generated large amounts of R*, which are then phosphorylated by GRK1. Surprisingly, phosphorylated species of rhodopsin persisted much longer in retinae of *Arr1*^−/−^ mice (>3 h) than WT C57 mice (∼1 h). This result cannot be attributed to an indirect effect of cell stress because of prolonged transducin signaling (Wang and Chen, 2014), because delayed dephosphorylation persisted in the *Arr1*^−/−^*Gnat1*^−/−^ double KO mice. Additionally, persistent rhodopsin phosphorylation in *Arr1*^−/−^ retinae cannot be explained by a reduction of protein phosphatase 2A (PP2A), the putative rhodopsin phosphatase. The ability of visual arrestin to promote rhodopsin dephosphorylation correlated with its ability to bind to R*P: rods expressing cone arrestin (ARR4), which binds R*P poorly (Chan et al., 2007), showed a similar time course of rhodopsin dephosphorylation as *Arr1*^−/−^ mice, whereas rods expressing the binding competent mutant, ARR1-3A (Song et al., 2009), showed a similar time course as WT mice. Together, the data suggest a novel role of ARR1 in facilitating rhodopsin dephosphorylation *in vivo*.

## Materials and Methods

### Genetically modified mouse lines

The use of mice in these experiments was in accordance with the guidelines established by the National Institutes of Health and the Animal Care and Use Committee of University of Southern California. Both male and female mice were used. C57 mice were purchased from The Jackson Laboratory. Generation of *Arr1*^−/−^ (Xu et al., 1997), *Arr4*^*Arr1*−/−^ (Chan et al., 2007), and *Arr1-3A*^*Arr1*−/−^ (Song et al., 2009; Samaranayake et al., 2018) transgenic mice and their genotyping protocols were previously described. C57 mice and *Arr1-3A* transgenic mice were maintained at 12 h light/12 h dark cycle, whereas *Arr1*^−/−^ and *Arr4*^*Arr1*−/−^ transgenic mice were born and raised in darkness to avoid light-induced retinal degeneration (Chen et al., 1999). Mice were dark-adapted overnight, and retinae were dissected under infrared light for the dark condition. For light exposure, pupils of dark-adapted mice were dilated with 0.5% tropicamide and 2.5% phenylephrine hydrochloride ophthalmic solutions (Akorn) before they were placed in a clear cage and exposed to 5000 lux light for 15 min. Retinae were immediately dissected (T = 0), or mice were returned to darkness and their retinae were isolated under infrared light after the indicated times. Retinae were snap frozen in liquid N_2_ and stored in −80°C until further use.

### Detection of differently phosphorylated species of rhodopsin by isoelectric focusing (IEF)

Detailed methods of IEF and rhodopsin detection have been described (Lokappa et al., 2019). Briefly, frozen retinae were thawed on ice and homogenized in buffer [25 mm HEPES, pH 7.5, 100 mm EDTA, 50 mm NaF, 5 mm adenosine, protease inhibitors cocktail (Roche Life Science)] using a Polytron. Membrane pellet was collected by centrifugation (13,000 × *g*, 4°C) for 15 min. Pellets were washed 3 times with 10 mm HEPES buffer, pH 7.5, and resuspended in regeneration buffer (10 mm HEPES, pH 7.5, 1 mm MgCl_2_, 0.1 mm EDTA, 2% BSA, 50 mm NaF, 5 mm adenosine, protease inhibitor cocktail) with 3-4 molar excess of 11-*cis* retinal over rhodopsin and incubated overnight at 4°C with gentle mixing to regenerate rhodopsin. Membrane pellets were again collected by centrifugation, washed twice with HEPES buffer, and dissolved in solubilization buffer (10 mm HEPES, pH 7.5, 1 mm MgCl_2_, 0.1 mm EDTA, 1% dodecyl-maltoside, 1 mm DTT) overnight at 4°C. The samples were centrifuged (4°C, 5 min at 19,000 × *g*) to remove particulates. Supernatants were loaded onto polyacrylamide IEF gel with pH range 2.5-8, as described (Lokappa et al., 2019). IEF was performed in darkness, and the proteins were transferred onto a nitrocellulose membrane by capillary action. Rhodopsin was detected by Western blot using either R2-12N (Adamus et al., 1991) or 4D2 (Laird and Molday, 1988) primary antibodies, both of which bind the aminoterminus of rhodopsin.

### Rhodopsin quantification

Mice were treated to the same lighting protocol as described above. All the subsequent steps were performed under dim red light, and the samples protected from light by wrapping the tubes in aluminum foil. Isolated retinae were each placed in 200 μl PBS containing 1% dodecyl-maltoside and incubated at 4°C with gentle agitation for 3 h to dissolve the tissue. The samples were centrifuged (5 min, 19,000 × *g*) to remove particulates, and absorption spectra (300-600 nm) were recorded. After recording the dark spectra, the samples were fully bleached by exposing to bright light for 2 min, and the difference in absorption at 500 nm was used to calculate the amount of rhodopsin based on its absorption coefficient (40,600 m^−1^ cm^−1^).

### Immunocytochemistry

The cornea and lens were removed and the remaining eyecup fixed with 4% formaldehyde in PBS for 15 min on ice. The tissues were rinsed 3 times in PBS, placed in 30% sucrose overnight at 4°C, embedded in OCT (Tissue Tek, Electron Microscopy Science), and 10 μm frozen sections were obtained. Tissue sections were blocked for 1 h in PBS containing 1% horse serum and 0.1% Triton X-100. Sections were then incubated with antibody against ARR1 (1:200 dilution) (Chen et al., 2006; Rose et al., 2017) and rhodopsin (1D4, 1:1000) (MacKenzie et al., 1984), followed by 1 h incubation with a secondary anti-mouse antibody (#TI-2000, Vector Laboratories). Images were obtained by Zeiss Axioscope 2 using the same exposure time for each antibody.

### Western blots

Retinae were isolated and homogenized in buffer [80 mm Tris, pH 8.0, 4 mm MgCl_2_, and 0.5 mg/ml protease inhibitor cocktail (Roche)] and centrifuged at 30,000 × *g* for 10 min. The supernatant was collected (soluble fraction), and the pellet was solubilized using Triton X-100-containing buffer [80 mm Tris, pH 8.0, 4 mm MgCl_2_, 1% Triton X-100, and 0.5 mg/ml protease inhibitor cocktail (Roche)]. Protein was quantified using Bradford protein assay (Bio-Rad). Equal amount was loaded per lane and separated in 12% Bis-Tris SDS-PAGE gel (Invitrogen) and transferred onto nitrocellulose membranes. The blots were blocked in TBS-T buffer (20 mm Tris, pH 7.5, 136.8 mm NaCl, and 0.1% Tween 20) containing 5% nonfat dry milk. The following primary antibodies were used: anti-PP2A A subunit (1:500, #2041, Cell Signaling), anti-PP2A C subunit (1:2000, #2259, Cell Signaling), and rabbit polyclonal anti-Gβ5 (1:2000) (Watson et al., 1996). The secondary antibodies IRDye 680 goat anti-mouse and IRDye 800 goat anti-rabbit antibodies (LI-COR Biosciences) were used. The proteins were visualized and quantified using Odyssey Infrared Imaging System (LI-COR Biosciences).

### Rod outer segment (ROS) isolation

Isolation of ROSs was performed as described (Moaven et al., 2013). Ten retinae were collected from mice with indicated genotypes. To detach the outer segments, retinae were vortexed for 2 min in 120 µl of 8% OptiPrep/Ringer's buffer (OptiPrep density gradient medium, Sigma; Ringer's: 130 mm NaCl, 3.6 mm KCl, 2.4 mm MgCl_2_, 1.2 mm CaCl_2_, 10 mm HEPES, 0.02 mm EDTA, pH 7.4, osmolarity at 313 mosM). After a quick spin at 510 × *g* for 1 min, 100 µl of supernatant was taken out and another 100 µl of 8% OptiPrep buffer was added. Pellets were vortexed again, and the process repeated for 5 times to collect 500 µl of supernatant. The supernatant was loaded to the top of an OptiPrep gradient made by mixing 1.4 ml of 10% OptiPrep/Ringer's buffer with 1.5 ml of 18% OptiPrep/Ringer's buffer. Samples were centrifuged at 70,000 rpm (Beckman TLA100), 4°C, for 1 h. The ROS band was removed, and 3× volume of chilled Ringer's buffer was added. Samples were centrifuged again at 50,000 rpm (Beckman TLA100), 4°C, for 20 min. This process was repeated, and the pellets were kept at −80°C for later use.

### Statistics

Signal intensities of Western blots were quantified using the ImageJ software. Values are mean ± SD. Significance of the differences between genotypes was determined by one-way ANOVA. If the value reached significance, pairwise *post hoc* Tukey HSD test was performed.

## Results

### Rhodopsin dephosphorylation is delayed in retinae of *Arr1*^–/–^ mice

**Figure 1.**
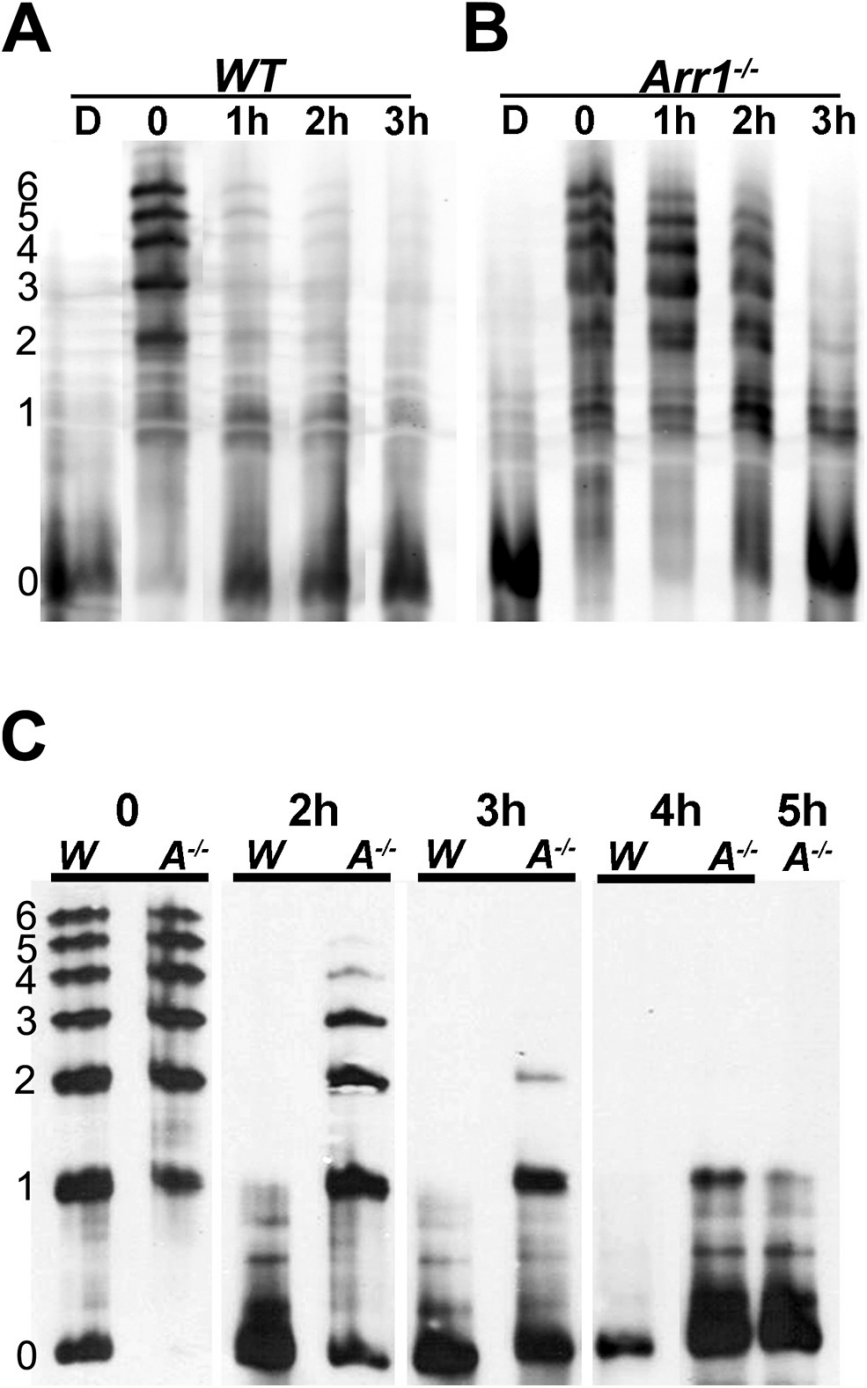
Rhodopsin dephosphorylation is delayed in retinae of *Arr1*^−/−^ mice. Differently phosphorylated species of rhodopsin were separated by IEF followed by Western blotting. Left, The number of phosphates on rhodopsin. ***A***, WT and (***B***) *Arr1*^−/−^ mice were dark-adapted (D) or exposed to light and returned to darkness for the indicated times (0-3 h). Immediately after light exposure (0), most of the rhodopsin shifted to phosphorylated species (1-6 phosphates). Levels of the phosphorylated species were greatly reduced in WT, but not *Arr1*^−/−^, after 1 h of dark adaptation. ***C***, Comparison of WT (W) and *Arr1*^−/−^ (A^−/−^) rhodopsin phosphorylation status on a longer time scale (0-5 h).

The presence of ARR1 interfered with rhodopsin dephosphorylation in isolated bovine ROSs (Palczewski et al., 1989b; Azarian et al., 1995). To test whether ARR1 delays rhodopsin dephosphorylation *in vivo*, control C57/B6 mice and *Arr1*^−/−^ mice were exposed to light followed by different periods of dark adaptation to compare the status of rhodopsin phosphorylation (Fig. 1). Mouse rhodopsin contains three serine and three threonine potential phosphorylation sites at its carboxylterminus (al-Ubaidi et al., 1990) that are substrates for GRK1. Thus, mouse rhodopsin can contain up to 6 phosphates. The differently phosphorylated species of rhodopsin were separated based on their isoelectric points in an acrylamide gel with a pH gradient. After transferring the proteins onto nitrocellulose, rhodopsin was visualized using an antibody against its amino terminus to detect all phosphorylated species (Lokappa et al., 2019). In the dark-adapted retina, rhodopsin is unphosphorylated (0P, D for dark adapted retina, Fig. 1*A*,*B*). Following bright light exposure, rhodopsin shifted to multiply phosphorylated forms, the majority of which contained 2P to 6P (Fig. 1*A*,*B*). In WT mice, most of rhodopsin molecules became dephosphorylated after 1 h in darkness (Fig. 1*A*), consistent with previous observations in mouse rods (Ohguro et al., 1995; Kennedy et al., 2001). Surprisingly, rhodopsin remained highly phosphorylated in the *Arr1*^−/−^ retina following 1 or 2 h of dark adaptation; albeit a shift toward lower phosphorylated species was observed (Fig. 1*B*). After 3 h in darkness, some monophosphorylated species remained (Fig. 1*B*). To examine the time course necessary for the completion of rhodopsin dephosphorylation in the *Arr1*^−/−^ retina, mice were exposed to bright light and returned to darkness for 2, 3, 4, and 5 h (Fig. 1*C*). Again, rhodopsin dephosphorylation was nearly complete by 2 h in darkness in the WT retina, whereas a trace amount of 1P species remained in the *Arr1*^−/−^ retina even after 5 h in darkness (Fig. 1*C*). These results show that, in contrast to *in vitro* results, the absence of ARR1 delayed rhodopsin dephosphorylation *in vivo*.

### Delayed rhodopsin dephosphorylation in retinae of *Arr1*^−/−^ mice is not caused by persistent signaling-induced cellular stress or differences in levels of regenerated rhodopsin

**Figure 2.**
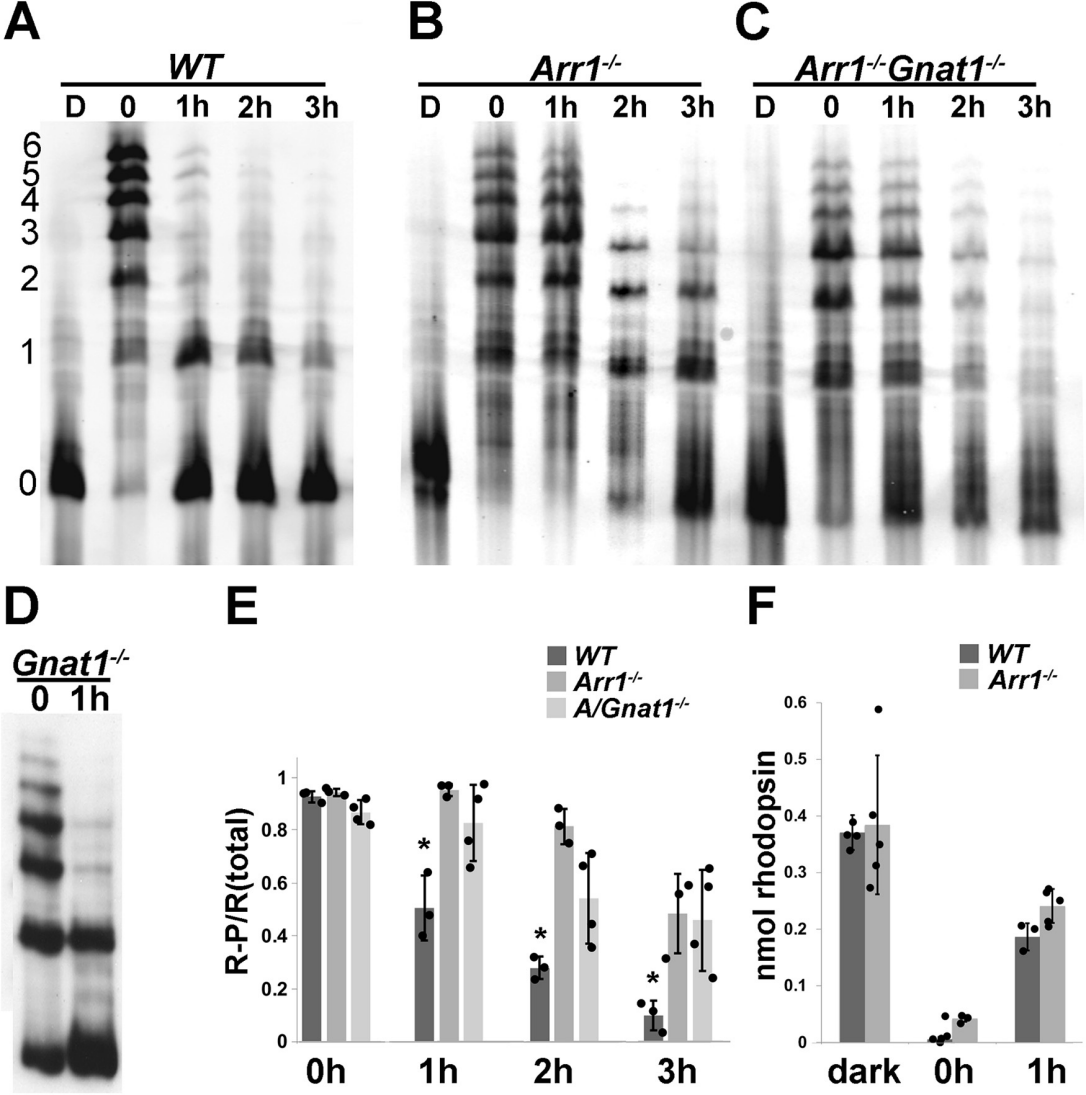
Delayed rhodopsin dephosphorylation in retinae lacking ARR1 is not because of cell stress caused by persistent transducin signaling. The status of rhodopsin phosphorylation was examined using IEF in (***A***) WT mice, (***B***) *Arr1*^−/−^ mice, (***C***) *Arr1*^−/−^*Gnat1*^−/−^ mice, and (***D***) *Gnat1*^−/−^ mice. ***E***, Ratio of signals summed from all phosphorylated species (1-6) and all rhodopsin species (0-6 phosphates) as a function of time. Values indicate mean ± SD; *N* ≥ 3. One-way ANOVA and *post hoc* Tukey's HSD showed no difference at 0 h, but a significant difference between WT and *Arr1*^−/−^ and *Arr1*^−/−^/*Gnat1*^−/−^ samples at 1, 2, and 3 h (*p* < 0.01). ***F***, Quantification of the amount of total rhodopsin present per retina in dark-adapted (dark) and mice immediately after light exposure (0) and after 1 h in darkness (mean ± SD; *N* ≥ 3).

The absence of ARR1 leads to persistent activation of the visual G-protein, transducin, by R* (Xu et al., 1997). This, in turn, induces the unfolded protein response followed by cell death (Wang and Chen, 2014). Consistent with transducin-mediated mechanism of cell death caused by light exposure, the stress response and retinal degeneration were prevented when the *Arr1*^−/−^ mice were crossed into the transducin KO (*Gnat1*^−/−^) background (Hao et al., 2002; Wang and Chen, 2014). To test whether the delay in rhodopsin dephosphorylation in the *Arr1*^−/−^ retina is a nonspecific effect of cellular stress, we exposed *Arr1*^−/−^ and *Arr1*^−/−^*Gnat*^−/−^ double KO mice to bright light, followed rhodopsin's phosphorylation status after different times in darkness, and compared the time course of dephosphorylation with WT mice (Fig. 2). In all genotypes, nearly all rhodopsin molecules became phosphorylated immediately following light exposure. Consistent with the results shown in Figure 1, most rhodopsin molecules were dephosphorylated after 3 h of dark adaptation in WT mice (Fig. 2*A*). In contrast, phosphorylated species of rhodopsin persisted even after long times of dark adaptation in both *Arr1*^−/−^ (Fig. 2*B*) and *Arr1*^−/−^*Gnat1*^−/−^ mice (Fig. 2*C*). This delay was not caused by the absence of transducin, since rhodopsin was efficiently dephosphorylated in *Gnat1*^−/−^ mice, similar to WT mice (Fig. 2*D*). To quantify these results, the band intensities of unphosphorylated rhodopsin, as well as different species of phosphorylated rhodopsin in the IEF gels were measured using ImageJ and summed, and the ratio of phosphorylated rhodopsin (1P to 6P) to total rhodopsin was plotted as a function of time (Fig. 2*E*, mean ± SD, *N* ≥ 3). In all three groups, the population of rhodopsin molecules was equally phosphorylated immediately following light exposure, indicating similar GRK1 activity (Fig. 2*E*, T = 0 h). In the WT retina, the fraction of phosphorylated rhodopsin steadily decreased during 1-3 h of dark adaptation, whereas the fraction of R-P remained elevated for both *Arr1*^−/−^ and *Arr1*^−/−^*Gnat1*^−/−^ retinae (Fig. 2*E*). A one-way ANOVA with *post hoc* Tukey test revealed significant differences in the 1, 2, and 3 h time points between WT and *Arr1*^−/−^ and *Arr1*^−/−^*Gnat1*^−/−^ mice, but no differences between *Arr1*^−/−^ and *Arr1*^−/−^*Gnat1*^−/−^ mice. It has been suggested previously that rhodopsin dephosphorylation may require pigment regeneration (Kennedy et al., 2001). To test whether pigment regeneration accounts for the difference in the rate of rhodopsin dephosphorylation, we compared the amount of regenerated rhodopsin between retinae from WT and *Arr1*^−/−^ mice before (Fig. 2*F*, dark), immediately after light exposure, and after 1 h of dark adaptation (Fig. 2*F*, 0 and 1, respectively). Baseline quantity of rhodopsin in dark-adapted mice was similar in both WT and *Arr1*^−/−^ retinae. Light exposure caused a near-complete bleach of all rhodopsins, consistent with the IEF results that nearly all rhodopsin molecules were activated and became phosphorylated on light exposure. After 1 h in darkness, the amount of regenerated rhodopsin was similar in WT and *Arr1*^−/−^ retinae (Fig. 2*E*). Thus, the different rate of rhodopsin dephosphorylation cannot be attributed to an indirect effect of rhodopsin regeneration. These results are consistent with the notion that ARR1 is required for timely rhodopsin dephosphorylation *in vivo*.

### Time course of rhodopsin dephosphorylation correlates with ARR1 translocation

**Figure 3.**
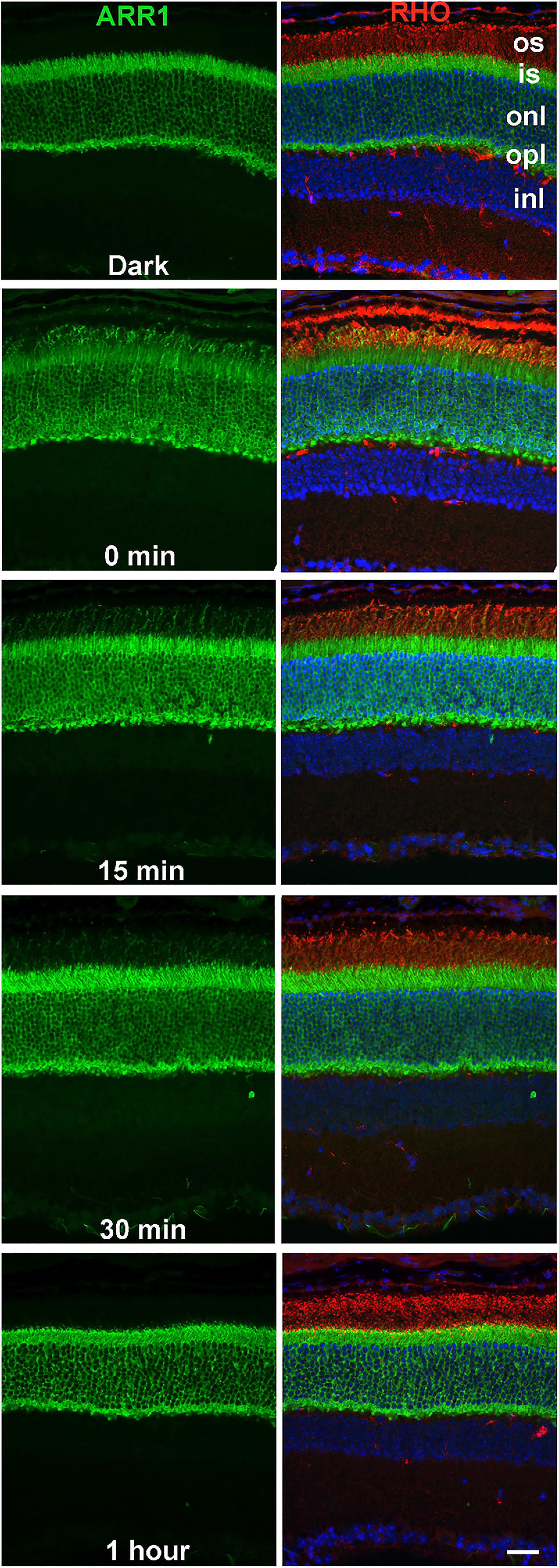
Light induced ARR1 translocation to the outer segment, and its return to the inner segments in the dark has the same time course as rhodopsin dephosphorylation. ARR1 is visualized by immunofluorescence (green) in frozen retinal sections. Left panels represent only ARR1. Right panels also represent superimposed rhodopsin labeling (red) to visualize the location of the outer segment. Nuclei are labeled with DAPI (blue). os, Outer segment; is, inner segment; onl, outer nuclear layer; opl, outer plexiform layer; inl, inner nuclear layer. Scale bar, 20 μm.

In the dark-adapted retina, ARR1 is sequestered in the inner segment compartments (Whelan and McGinnis, 1988; Strissel et al., 2006). Upon light exposure, ARR1 diffuses to the outer segment to bind its high-affinity target, R*-P (Nair et al., 2005). Given that ARR1 facilitates rhodopsin dephosphorylation, we examined whether ARR1's movement toward the outer segment correlates with the timing of rhodopsin dephosphorylation under our experimental conditions. Consistent with previous observations, ARR1's immunoreactivity is predominantly in the cytoplasmic space of the inner segment, outer nuclear, and outer plexiform layers of dark-adapted retina (Fig. 3, Dark). Following 15 min of bright light exposure, ARR1 immunoreactivity was detected in the outer segment (Fig. 3, 0 min). This fluorescent signal at the outer segment progressively weakened after 15 and 30 min of dark adaptation and returned to the dark-adapted baseline pattern after 1 h in darkness (Fig. 3). The timing of this movement corresponds well to the timing of rhodopsin dephosphorylation (Fig. 2) and supports a potential role for ARR1 in mediating rhodopsin dephosphorylation in intact rods.

### *Arr1*^−/−^ and WT ROSs contain similar levels of PP2A

**Figure 4.**
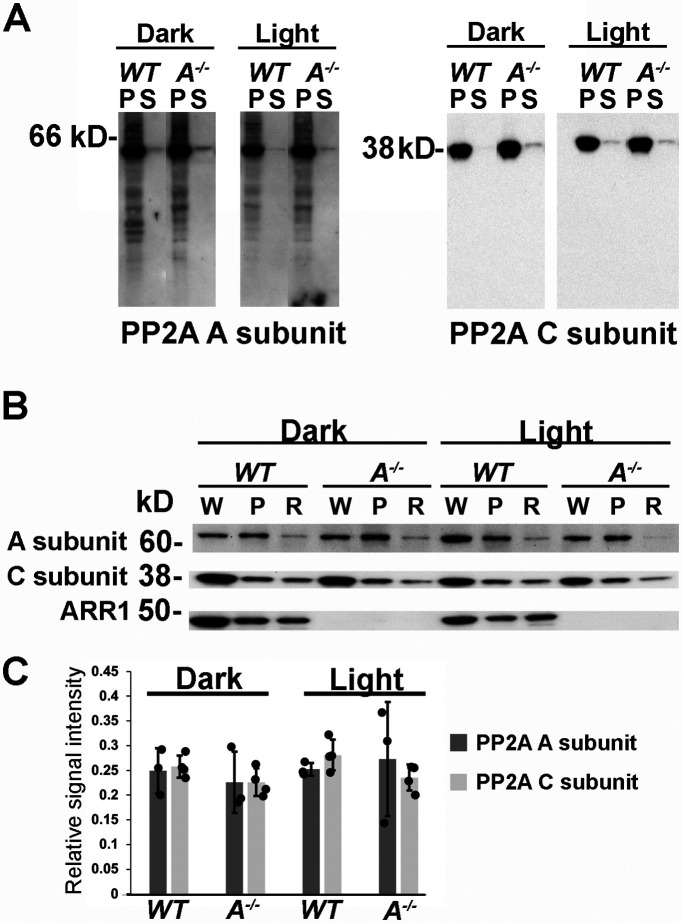
PP2A levels and localization are not affected by ARR1 or light exposure. ***A***, Western blot of membrane (P) and soluble (S) fractions of retinal homogenates from dark-adapted or light-exposed WT and *Arr1*^−/−^ mice probed with antibodies against both isoforms of the A subunits or the C subunits. Equal amounts of proteins were loaded per lane. ***B***, Western blots of whole retinal homogenate (W), the membrane fraction (P), or ROS (R) from dark-adapted or light-exposed WT or *Arr1*^−/−^ mice probed with the indicated antibodies. ***C***, Quantification of signals from PP2A A (*N* = 3) and C subunits (*N* = 4) in ROSs isolated from dark-adapted or light-exposed WT and *Arr1*^−/−^ mice.

Rhodopsin dephosphorylation *in vitro* can be catalyzed by several different phosphatases (Yang et al., 1991; Kutuzov and Pfister, 1994; Kutuzov and Bennett, 1996; Klumpp et al., 1998), including PP2A (Fowles et al., 1989; Palczewski et al., 1989a; King et al., 1994). Conditional KO of a catalytic subunit of PP2A (Cα) in mouse rods resulted in delayed rhodopsin dephosphorylation, supporting a functional role of PP2A as the pigment phosphatase *in vivo* (Kolesnikov et al., 2017). PP2A holoenzyme is composed of three subunits: the catalytic subunit C, the scaffolding subunit A, and the regulatory subunit B. Two isoforms of the catalytic subunits, Cα and Cβ (Mustafi et al., 2012), and two isoforms of the A subunits, Aα and Aβ (Liu et al., 2008), are expressed in the retina. To investigate whether the absence of ARR1 affected rhodopsin dephosphorylation through altering PP2A levels or localization, we took advantage of antibodies that recognize both isoforms of the C subunit and antibodies that bind both isoforms of the A subunit, thereby assessing all PP2A isoforms present in the sample. Western blots were performed in retinal extract from dark-adapted and light-exposed mice. Retinal extracts were separated into membrane (P) and soluble fractions (S) to investigate whether the presence of ARR1 or light exposure affects the distribution of PP2A to the membrane fraction that contains rhodopsin (Fig. 4*A*). Both the A and C subunits levels were similar in WT and *Arr1*^−/−^ samples, and both subunits were associated with the membrane fraction independent of light conditions (Fig. 4*A*). To examine the level of PP2A in the ROSs where rhodopsin is localized, ROSs were isolated from retinae of dark-adapted and light-exposed mice. A comparison of A and C subunit levels between whole retinal extract (W), the membrane fraction (P), and ROS (R) shows relatively low PP2A expression in ROS (Fig. 4*B*). To quantify PP2A levels in ROS and to examine any light- or ARR1-dependent changes in this compartment, Western blots were performed on purified ROS from dark-adapted and light-exposed WT and *Arr1*^−/−^ mice. Results from Figure 4*C* show similar levels of A and C subunits of PP2A in WT and *Arr1*^−/−^ ROS and no effect of light exposure on the levels of these proteins. Collectively, these data show that ARR1's impact on rhodopsin dephosphorylation is not through altering PP2A levels or its localization.

### Cone arrestin does not facilitate rhodopsin dephosphorylation

**Figure 5.**
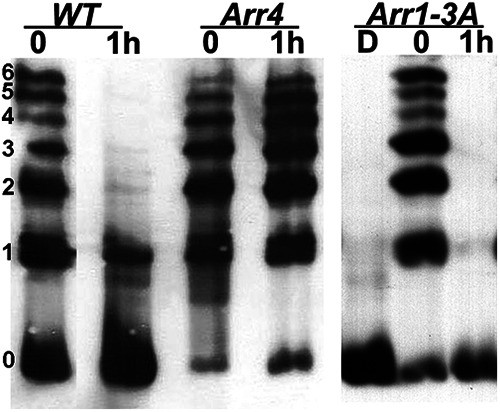
Rhodopsin dephosphorylation in transgenic mice expressing different visual arrestins in rods. Representative image from three independent experiments. C57 WT and mice that express the ARR4 or ARR1-3A transgenes on the *Arr1*^−/−^ background were exposed to light and returned to darkness for 1 h. The status of rhodopsin phosphorylation was examined by IEF. Left, The number of rhodopsin-attached phosphates.

We next examined the ability of different forms of arrestins in promoting rhodopsin dephosphorylation. Rods express ARR1, whereas cones express both ARR1 and cone arrestin (ARR4) (Nikonov et al., 2008). We had previously compared functional differences between ARR1 and ARR4 in deactivating R*-P by expressing ARR4 in rod photoreceptors of *Arr1*^−/−^ mice (Chan et al., 2007). ARR4 exhibited similar light-induced translocation as ARR1 in rods and exerted a reduction of R*-P's catalytic activity compared with that of *Arr1*^−/−^ light responses. However, ARR4 was not able to fully terminate R*-P's activity. Instead, the light response reached a lower steady state, and full recovery occurred only on Meta II rhodopsin (MII) decay (Chan et al., 2007). The activity of ARR4 toward R*-P resembles that of rapid on-off, low-affinity interaction that reduced the efficiency of transducin activation but did not fully prevent it (Chan et al., 2007). As shown in Figure 5, light exposure led to the formation of multiply phosphorylated species of rhodopsin in both WT and ARR4-expressing transgenic mice, demonstrating normal access of GRK1 to its substrate. After 1 h of dark adaptation, most of the rhodopsin molecules have been dephosphorylated in the WT, but not in the *Arr4*^*Arr1*−/−^ retina (Fig. 5). Thus, the phenotype of the *Arr4*^*Arr1*−/−^ retina resembles that of *Arr1*^−/−^ with respect to rhodopsin dephosphorylation. We also investigated a transgenic line that expressed the 3A ARR1 mutant in the *Arr1*^−/−^ rods (ARR1-3A). ARR1-3A is a gain-of function mutant that demonstrates high binding to light-activated unphosphorylated rhodopsin, R* as well as R*-P because of the triple alanine substitutions in its carboxylterminus (Vishnivetskiy et al., 2000). These substitutions disrupt one of the intramolecular constraints that maintain ARR1 in the inactive, basal conformation. Consequently, the ARR1-3A mutant can be converted to a binding-competent form by R* (Vishnivetskiy et al., 2000). Like WT and *Arr4*^*Arr1*−/−^ retinae, rhodopsin was efficiently phosphorylated in *Arr1-3A*^*Arr1*−/−^ retinae on light exposure (Fig. 5), indicating that the enhanced ability of ARR1-3A to bind R* did not interfere with GRK1's access to R* under our experimental conditions. After 1 h in darkness, the majority of rhodopsin molecules has been dephosphorylated, similar to WT (Fig. 5). These results show that the ability of visual arrestin to promote rhodopsin dephosphorylation correlated with its ability to bind R*-P.

## Discussion

After light activation, rhodopsin molecules must return to the ground state to regain their ability to initiate phototransduction. Removal of phosphates on the rhodopsin's carboxylterminus is a key step in this process because a phosphorylated, 11-*cis* retinal bound rhodopsin, when photolyzed, generates a smaller quantal response (Berry et al., 2016). This, in turn, would reduce sensitivity and affect signal transfer at the rod-to-rod bipolar cell synapse (Sampath and Rieke, 2004; Field and Sampath, 2017). In contrast to our knowledge of rhodopsin phosphorylation which is catalyzed by GRK1 (Kuhn, 1978; Arshavsky, 2002), much less is known about how rhodopsin is dephosphorylated. The central finding of our work is that ARR1 is required for efficient rhodopsin dephosphorylation *in vivo*. This finding was unexpected because *in vitro* experiments using purified bovine ROSs show that ARR1's binding to R*-P inhibited rhodopsin dephosphorylation (Palczewski et al., 1989b; Azarian et al., 1995). We explored the possibility that the delayed dephosphorylation in *Arr1*^−/−^ rods is an indirect effect of light-induced cell stress caused by persistent transducin signaling. Indeed, we have shown previously that amplified phototransduction in the *Arr1*^−/−^ triggers endoplasmic reticulum stress and activation of the unfolded protein response, leading to rod death (Wang and Chen, 2014). Consistent with a causal role of transducin signaling in this light damage model, unfolded protein response stress and cell death were prevented when *Arr1*^−/−^ mice were crossed into the *Gnat1*^−/−^ background (Wang and Chen, 2014). If the delayed rhodopsin dephosphorylation in *Arr1*^−/−^ mice is an indirect effect of cell stress, this effect should be circumvented in the *Arr1*^−/−^*GnaT1*^−/−^ double KO mice. However, we observed a similar slow rate of rhodopsin dephosphorylation in the *Arr1*^−/−^ and *Arr1*^−/−^*Gnat1*^−/−^ mice. This result suggests a direct and novel role of ARR1 in promoting rhodopsin dephosphorylation (Fig. 2). We also observed that rhodopsin dephosphorylation in *Gnat1*^−/−^ mice expressing normal complement of ARR1 was similar to that of WT mice (Fig. 2), demonstrating that rhodopsin dephosphorylation does not depend on transducin signaling. Because transducin signaling leads to a drop in Ca^2+^ concentration in the outer segment (Woodruff et al., 2002), our data suggest that the activity of the rhodopsin phosphatase is not regulated by this change in Ca^2+^ concentration.

PP2A is thought to be rhodopsin's phosphatase in mammalian rods based on early pharmacologic studies on isolated ROSs using phosphatase inhibitors (Fowles et al., 1989; Palczewski et al., 1989a; King et al., 1994). More recently, the role of PP2A in rhodopsin dephosphorylation *in vivo* was supported by evidence from a conditional KO of a catalytic subunit, Cα, of PP2A in mouse rods, which showed that rhodopsin dephosphorylation was delayed, although not abolished (Kolesnikov et al., 2017). This delay may be because of an incomplete ablation of PP2A because the other catalytic subunit isoform, Cβ, is also expressed in the retina (Mustafi et al., 2012). In addition to the catalytic C subunit, the PP2A holoenzyme contains a scaffold A subunit and a regulatory B subunit. The A and C subunits are each encoded by two different genes that generate two isoforms of high-sequence identity. On the other hand, the regulatory B subunits are very diverse and are subdivided into four families, each of which contains several different genetically encoded isoforms (Sents et al., 2013; Sandal et al., 2021). The multiplicity of these subunits allows for many combinatorial possibilities with diverse function in different cells and makes it challenging to identify which specific isoform is responsible for a particular functional task. We used antibodies that bind common epitopes on both catalytic C isoforms (Cα and Cβ) and antibodies that recognize both scaffold isoforms (Aα and Aβ) to test whether the absence of ARR1 lowered overall PP2A levels. Because ARR1 becomes membrane bound on binding to R*P, we also investigated the distribution of PP2A between membranes and cytosol and whether this distribution differed in dark-adapted and light-exposed retinae (Fig. 4). We saw no changes in PP2A levels in the *Arr1*^−/−^ retinae and found that PP2A is membrane-associated independently of light exposure. We found that the level of PP2A in ROS was very low compared with that of whole retinal extract or the membrane fraction, consistent with previous findings (Rajala et al., 2018), and also consistent with low specific phosphatase activity in the ROS (Palczewski et al., 1989b). Again, no differences in PP2A levels in ROSs were observed between WT and *Arr1*^−/−^ retinae in the dark or on light exposure. Future experiments aimed at determining the exact subunit composition of PP2A for rhodopsin dephosphorylation, and the ratio of rhodopsin to PP2A may yield insights into whether PP2A alone can sustain observed rapid rhodopsin dephosphorylation *in vivo*.

The reconstitution of the visual pigment by the incorporation of 11-*cis* retinal supplied by the RPE is a slow process with a time course like that of rhodopsin dephosphorylation, both potentially affecting full recovery of rod sensitivity in the dark. Conditional KO of PP2A Cα slowed the conversion of all-trans retinal to all-trans retinol in the visual cycle (Kolesnikov et al., 2017). We investigated whether rhodopsin regeneration was altered in *Arr1*^−/−^ mice and found a similar quantity of regenerated rhodopsin in WT and *Arr1*^−/−^ mice after 1 h of dark adaptation. This is consistent with previous experiments using isolated eyecups from *Arr1*^−/−^ mice that showed little difference in the reduction of all-trans retinal to all-trans retinol, or other intermediates in the visual cycle, compared with WT mice (Palczewski et al., 1999). Thus, a difference in pigment regeneration does not explain slowed rhodopsin dephosphorylation in *Arr1*^−/−^ mice. This conclusion is also supported by findings in carp rods and cones where dephosphorylation of phosphorylated visual pigments and phosphorylated opsin apoproteins occurs with similar time course (Yamaoka et al., 2015).

Although ARR4 exhibits the same light-induced movement toward the outer segment as ARR1 in rods (Chan et al., 2007), it was not able to facilitate rhodopsin dephosphorylation (Fig. 4). We had previously shown that ARR4 does not fully terminate the catalytic activity of R*-P, indicating a lack of high-affinity binding (Chan et al., 2007). Thus, high-affinity interaction between ARR1 and R*P may be required to facilitate rhodopsin dephosphorylation. Indeed, the mutant, ARR1-3A, which has enhanced ability to bind both R*-P and R*, promoted rhodopsin dephosphorylation normally. The inability of ARR4 to promote rhodopsin dephosphorylation raises the interesting possibility that ARR1, also expressed in cones at very high levels (Nikonov et al., 2008), may facilitate dephosphorylation of cone opsins.

As mentioned above, the 15 amino acid element at the carboxylterminus of mouse rhodopsin contains 6 Ser and Thr sites that are substrates for GRK1. The last 5 residues, QVAPA, contain a trafficking signal necessary for rhodopsin to travel from the endoplasmic reticulum to the ROS (Concepcion et al., 2002; Concepcion and Chen, 2010). Evidence that ARR1 binding masks the phosphates is supported by our studies on a constitutively active rhodopsin mutant, K296E (Chen et al., 2006; Moaven et al., 2013), which is stably bound to ARR1 (Li et al., 1995), as well as by the structure of the rhodopsin-ARR1 complex (Zhou et al., 2017). ARR1 binding masked the trafficking signal and caused K296E to mislocalize to the cell body. This mislocalization is corrected when K296E is expressed on the *Arr1*^−/−^ background, whereupon K296E became localized to ROS (Chen et al., 2006). All available data suggest that the nearby phosphates are also masked by ARR1 binding. Extensive mutagenesis (for review, see Gurevich and Gurevich, 2004) and structural studies (Zhou et al., 2017) have identified several positively charged residues within ARR1 that bind phosphates attached to the receptor. These data are consistent with the idea that ARR1 binding to R*P shields the phosphorylated Ser and Thr from the phosphatase. How can these data, together with the *in vitro* evidence that ARR1 inhibits rhodopsin dephosphorylation (Palczewski et al., 1989b), be reconciled with the observed role of ARR1 in facilitating rhodopsin dephosphorylation *in vivo*? One possibility is that ARR1 may function as a scaffold to position the phosphatase to R*-P. Hydrolysis of all-trans retinal causes ARR1 to dissociate from the apoprotein opsin, allowing the phosphatase to access the phosphates on the carboxylterminus. Our results suggest that targeting of the phosphatase to R*-P is slowed in the absence of ARR1, leading to a delay, but not total absence of dephosphorylation in the *Arr1*^−/−^ rods. A close correlation in the time course for light-induced ARR1 translocation to the outer segment (Fig. 3) and opsin dephosphorylation (Fig. 1) is also consistent with a role of ARR1 in facilitating the positioning of the phosphatase toward R*-P. It may be of relevance that such a role for phosphatase recruitment has been observed for β-arrestin2, where it participates in the formation of signaling complexes containing PP2A and Akt with the D2 dopamine receptors (Beaulieu et al., 2005; Wu et al., 2020). Future studies to investigate ARR1-interacting partners will shed additional light on the molecular mechanism of this process.
